# Contract-Provident Medical Service

**Published:** 1911-05-20

**Authors:** 


### The Hospital

A Journal of

The Medical Sciences and Hospital Administration.

New Series. No. 224, Vol. IX. [No. 1296, Vol. L.] SATURDAY, MAY 20, 1911

### CONTRACT-PROVIDENT MEDICAL SERVICE.

Last week the then newly introduced Government scheme for dealing with sickness and invalidity by compulsory insurance was passed under review in these columns. That review was necessarily a very general one, and necessarily dealt very briefly with many of the most salient provisions of the Bill. From the point of view of the general practitioner there are certain aspects and clauses of the proposed legislation which affect him more nearly than others; and of these the conditions and organisation of the medical attention which is to be provided for the insured demand perhaps more careful and detailed scrutiny than any others. As yet there is no information, other than the very vaguest, available as to how this part of the scheme is to be worked. Fortunately, however, there was published on March 4 of this year a very full and admirable report of the Committee of the British Medical Association, appointed two years ago for the express purpose of considering the whole question of a "provident or insurance medical service." Whether Mr. Lloyd George has studied, or is likely to study, this report is, of course, impossible to say; but it is doing only bare justice to the work of the Committee to say that neither he nor any other Minister who tries to deal with the problems of national sickness and invalidity insurance can afford to neglect it. The authors of this report take always a broad view, and are mindful of the national welfare as well as of the purely personal interests of individual members of the medical profession; an admission which it gives the more pleasure to advertise inasmuch as the Association has in times past been sometimes criticised for acting too much in the narrow selfish spirit of industrial trade-unionism.

Within the limits here circumscribed it is as impossible to summarise the arguments and the conclusions of this Committee as it is to analyse fully the Insurance Bill itself. For all details, the Report itself, published as a supplement to the *British Medical Journal* for the date already mentioned, must be consulted. But it is of great interest to observe in how many respects the report foreshadows the proposals recently laid before Parliament, and how fully the effects of State medical insurance are discussed; and it is clear that an intimate acquaintance with the miserable conditions under which many overworked and underpaid members of the profession have to conduct contract practice has pervaded the Committee and influenced the report which has been issued. In the concluding section the Committee reviews the general effects of the development of an insurance medical service upon the interests of the public and the medical profession. The public advantages to be anticipated are improvement of the public health; inducement to poor people to contribute towards the cost of their own medical attendance, that is, to self-help; better application than at present of public funds for provision of medical attendance; and a more efficient medical service. The professional advantages similarly to be expected are greater prospect of adequate remuneration; improved general conditions of practice; and opportunities for doing better professional work. From the professional standpoint there are also certain disadvantages, such as disturbance of existing conditions which have proved relatively satisfactory to those concerned; and increased public interference between doctor and patient. It is also pointed out (paragraph 116)—and this is really one of the most important sentences in the report—that, in any attempt to calculate suitable rates under contemplated conditions, existing rates afford an imperfect basis for many reasons. Thus, women and children being admitted to the service, the existing inducement to practitioners to attend men at low rates, in the hope of obtaining private practice among their families, disappears. Existing rates, too, for women and children are entirely inadequate, and are practically a form of charity at the expense of the medical profession. Moreover, the sick-pay rolls of the Friendly Societies afford no guide to the amount of medical attendance required, because members frequently obtain medical services without declaring on their societies' funds for sick pay.

Then, too, the members of Friendly Societies are selected lives, whereas a service such as is now under consideration must include persons in every state of health. Here, indeed, is a consideration of immeasurable importance, because the scheme, as it is so far put forward, intends that the Friendly Societies shall have their work enormously extended, and that only those who do not join one or other of those organisations shall come within the province of the public medical service. This will mean that a large proportion of the *clientèle* of the latter will be the damaged lives of those who are refused by the societies; and the lot of those doctors who undertake to attend, for a State allowance which is sure to be on none too generous a scale, the mass of degenerates and chronically diseased among our lower-class population is one which, it is safe to say, will not be a very enviable one. This, then, is a part of the scheme whose evolution in Parliament must be most closely watched on behalf of the medical profession. The public, and members of Parliament, must be made to understand that sweating the profession is a policy which is sure to be disastrous in the long run, as it has always proved to be. To amend the miserable conditions of club practice is an aim which will benefit the people far more than the medical profession. If the Insurance Bill does that it will have achieved a great gain; but it will not do so, in our judgment, without criticism and expert revision, and these it must be the business of the medical profession to supply.

